# Epidemiological Survey and Characteristic Analysis of Hand Eczema Among Nursing Staff in Certain Regions of Xinjiang During the COVID-19 Pandemic: A Cross-Sectional Study

**DOI:** 10.7759/cureus.97209

**Published:** 2025-11-19

**Authors:** Qingjie Hu, Yan Han, Jin Zhang, Yu Yu, Xiaodong She, Fanhe Jiang, Meihua Fu

**Affiliations:** 1 Department of Allergy and Rheumatology, Hospital for Skin Diseases, Institute of Dermatology, Chinese Academy of Medical Sciences and Peking Union Medical College, Nanjing, CHN; 2 Center for Population Medicine and Global Health, Hospital for Skin Diseases, Institute of Dermatology, Chinese Academy of Medical Sciences and Peking Union Medical College, Nanjing, CHN; 3 Department of Dermatology, The Third People's Hospital of Xinjiang Uygur Autonomous Region, Urumchi, CHN; 4 Department of Allergy, Hospital for Skin Diseases, Institute of Dermatology, Chinese Academy of Medical Sciences and Peking Union Medical College, Nanjing, CHN; 5 Department of Mycology, Hospital for Skin Diseases, Institute of Dermatology, Chinese Academy of Medical Sciences and Peking Union Medical College, Nanjing, CHN

**Keywords:** atopic dermatitis, covid-19 pandemic, cross-sectional study, hand eczema, hand hygiene, incidence, nursing staff

## Abstract

Objective: The objective of the study is to investigate the epidemiological characteristics and influencing factors of hand eczema (HE) among nursing staff in certain regions of Xinjiang during the COVID-19 pandemic.

Methods: Nursing staff from one tertiary hospital, one secondary hospital, and 15 community hospitals in the Xinjiang Uyghur Autonomous Region were selected as subjects. Clinical data of all participants were collected using paper questionnaires from November 2022 to March 2023, and the incidence and influencing factors of HE were analyzed.

Results: A total of 218 (26.3%) cases of HE were identified. The incidence in surgical departments (30.6%) was higher than that in non-surgical departments (24.1%, χ^2^ = 3.951, p = 0.047). The incidence in the 20-30 age group (22.2%) was lower than that in the 31-40 age group (30.7%, χ^2^ = 6.57, p = 0.01) and the 51-60 age group (39.3%, χ^2^ = 4.29, p = 0.04). The incidence was significantly lower in the group with hand cleaning frequency of 0-5 times per day (12.1%) compared to the groups with 6-10 times per day (26.3%, χ^2^ = 5.095, p = 0.024), 11-20 times per day (25.9%, χ^2^ = 5.157, p = 0.023), and >20 times per day (29.9%, χ^2^ = 7.745, p = 0.005). Similarly, the incidence increased with the frequency of soap/hand sanitizer exposure. The incidence was lower in the group using moisturizer more than 10 times per day (15.5%) compared to those using it 3-5 times per day (27.0%, χ^2^ = 5.786, p = 0.016) and less than three times per day (36.3%, χ^2^ = 16.344, p < 0.001). No significant differences in HE incidence were found among groups divided based on the frequency of daily contact with alcohol-based hand rub or the duration of rubber glove use (p > 0.05). The incidence of allergic rhinitis, allergic conjunctivitis, asthma, metal allergy, and dry skin was higher in the HE group than in the non-HE group (p < 0.05). Logistic regression analyses showed that age, years of working experience, presence of allergic rhinitis, allergic conjunctivitis, metal allergy, daily handwashing frequency, and frequency of contacting soap/hand sanitizer were risk factors for developing HE, while moisturizer use was a protective factor.

Conclusion: The incidence of HE among nursing staff during the COVID-19 pandemic was relatively high and was associated with hand hygiene practices and the patients' atopic constitution. Enhanced skin care and health education should be implemented to reduce the occurrence of HE.

## Introduction

Hand eczema (HE) is a chronic inflammatory disease distributed on the hands and/or wrists, with a complex and diverse etiology and clinical manifestations. It has a significant occupational correlation and severely impacts the quality of life of patients. Risk factors for HE include wet exposure, atopic dermatitis (AD), early onset of HE, contact allergies, smoking, and cold/dry weather conditions [1]. HE is relatively common, and more than 50% of HE cases are associated with occupational exposure [2]. A recent systematic review and meta-analysis revealed a lifetime prevalence of 14.5%, a one-year prevalence of 9.1%, and a point prevalence of 4% of HE in the general population [3]. Nursing is one of the high-risk occupations for HE. Since the outbreak of the COVID-19 pandemic in 2019, nursing staff working in high-risk environments have experienced increased frequency of handwashing and prolonged use of protective gear, leading to a rise in the incidence of HE. The aim of this study is to investigate the incidence and related influencing factors of HE among nursing staff in Xinjiang during the COVID-19 pandemic and within three months after the end of the epidemic.

## Materials and methods

Study subjects

This cross-sectional study was based on a self-designed questionnaire survey (Appendices). Nursing staff from one tertiary hospital, one secondary hospital, and 15 community hospitals in the Xinjiang Uyghur Autonomous Region were selected as subjects. Clinical data were collected from November 2022 to March 2023. The study was approved by the Ethics Committee of the Hospital of Skin Diseases, Chinese Academy of Medical Sciences and Peking Union Medical College (Approval No.: 2022-063), and all participants provided informed consent.

Methods

Clinical data and questionnaire surveys were used to record information. The questionnaire was designed to capture a wide range of information, including (1) demographic information: age, gender, and years of working experience; (2) HE-related information: age at onset of HE, duration of the disease, skin rash characteristics, treatment history, and current disease status; (3) medical and allergic history: history of other medical conditions and allergic diseases, such as allergic rhinitis, asthma, and metal allergy; (4) hand hygiene practices: daily usage time of rubber gloves, frequency of handwashing and moisturizer usage, frequency of soap/hand sanitizer exposure, and types of detergents used; and (5) eczema-exacerbating factors: specific factors that participants believed worsened their eczema: such as soap, liquid soap, shampoo, and other personal hygiene products, detergents and other household cleaning and laundry products, food handling, working with wet hands, frequent handwashing, protective gloves, and so on. Participants with eczema were initially self-assessed based on provided diagnostic criteria. The criteria included symptoms such as erythema, itching, burning, stinging, dry skin with scales, cracks, exudation or crusting, tiny blisters, papules, and rapid appearance of itchy wheals or lumps. Self-assessments were then reviewed and confirmed by two experienced dermatologists to ensure accuracy.

Statistical analysis

Data were analyzed using SPSS 20.0 software (IBM Corp., Armonk, NY, US). Quantitative data were expressed as mean ± standard deviation, and differences between groups were compared using t-tests. Categorical data were presented as frequencies and percentages (%), and differences between groups were compared using chi-squared tests. Binary logistic regression analysis was used to identify factors influencing the occurrence of HE. Variables included in the regression model were selected based on their clinical relevance and statistical significance in the univariate analysis. The model was assessed for goodness of fit using the Hosmer-Lemeshow test. Odds ratios (OR) and 95% confidence intervals (CI) were calculated for each predictor variable. A p-value < 0.05 was considered statistically significant.

## Results

Clinical characteristics of patients

A total of 834 questionnaires were collected, with five incomplete ones excluded and 829 valid questionnaires. There were 26 (3.1%) men and 803 (96.9%) women, with a mean age of 31.6 ± 7.5 years and an average working experience of 9.3 ± 7.7 years. The department distribution included 279 (33.7%) in surgical departments and 550 (66.3%) in non-surgical departments. A total of 288 (34.7%) participants had other allergic diseases (e.g., asthma, allergic rhinitis, and allergic conjunctivitis) (Table 1).

**Table 1:** Demographic characteristics of the participants

Characteristics		N (%)
Gender	Male	26 (3.1)
	Female	803 (96.9)
Age categories	20-30	411 (49.6)
	31-40	303 (36.6)
	41-50	87 (10.5)
	51-60	28 (3.4)
Years of working	0-10	566 (68.3)
	11-20	182 (22.0)
	21-30	81 (9.8)
Working department	Surgical department	278 (33.5)
	Non-surgical department	551 (66.5)
Allergic diseases	Allergic rhinitis	198 (23.9)
	Allergic conjunctivitis	62 (7.5)
	Asthma	17 (2.1)
	Metal allergy	36 (4.3)

Incidence of HE

Among the 829 participants, 218 (26.3%) had HE, with 91 (41.7%) experiencing eczema on the wrists or forearms, presenting as erythema, papules, vesicles, fissures, dry skin with scales, itching, and burning pain. The incidence of HE in men (11.5%) was lower than in women (26.8%), but the difference was not statistically significant (χ^2^ = 3.02, p = 0.08). The incidence in surgical departments (30.6%) was significantly higher than in non-surgical departments (24.1%, χ^2^ = 3.951, p = 0.047). The age and work experience of the HE group were comparable to those of the non-HE group (p = 0.96 and p = 0.17, respectively). The incidence of HE varied across different age groups, with the 20-30 age group (22.2%) being significantly lower than the 31-40 age group (30.7%, χ^2^ = 6.57, p = 0.01) and the 51-60 age group (39.3%, χ^2^ = 4.29, p = 0.04) (Table 2).

**Table 2:** Comparison of HE incidence among 829 nursing staff ^a^Compared with the group with 0-5 times per day, p < 0.05; ^b^compared with the <10 times/day group, p < 0.05; ^c^compared with the <3 times/day group, p < 0.05. HE: hand eczema

	N (%)	HE group	Non-HE group	χ^2^	p-value
Gender					
Male	26 (3.1)	3 (11.5)	23 (88.5)	3.02	0.08
Female	803 (96.9)	215 (26.8)	588 (73.2)		
Age (year)					
20-30	411 (49.6)	92 (22.4)	319 (77.6)	0.069	0.027
31-40	303 (36.6)	93 (30.7)	210 (69.3)		
41-50	87 (10.5)	23 (26.4)	64 (73.6)		
51-60	28 (3.4)	11 (39.3)	17 (60.7)		
Working department					
Surgical department	278 (33.7)	85 (30.6)	193 (69.4)	3.951	0.047
Non-surgical department	551 (66.3)	133 (24.1)	418 (75.9)		
Dry skin					
Yes	105 (12.7)	62 (59.0)	43 (41.0)	66.537	<0.001
No	724 (87.3)	156 (21.5)	568 (78.5)		
Allergic diseases					
With allergic diseases	237 (28.6)	110 (46.4)	127 (53.6)	69.296	<0.001
Without allergic diseases	592 (71.4)	108 (18.2)	484 (81.8)		
Frequency of handwashing					
0-5 times/d	58 (7.0)	7 (12.1)	51 (87.9)	7.88	0.049
6-10 times/d	190 (22.9)	50 (26.3)^a^	140 (73.7)		
11-20 times/d	317 (38.2)	82 (25.9)^a^	235 (74.1)		
>20 times/d	264 (31.8)	79 (29.9)^a^	185 (70.1)		
Frequency of contact with soap/hand sanitizer					
<10 times/d	215 (25.9)	40 (18.6)	175 (81.4)	13.46	0.001
11-20 times/d	394 (47.5)	103 (26.1)^b^	291 (73.9)		
>20 times/d	220 (26.5)	75 (34.1)^b^	145 (65.9)		
Frequency of using alcohol-based hand sanitizer					
0-5 times/d	162 (19.5)	41 (25.3)	121 (74.7)	5.074	0.166
6-10 times/d	258 (31.1)	58 (22.5)	200 (77.5)		
11-20 times/d	214 (25.8)	57 (26.6)	157 (73.4)		
>20 times/d	195 (23.5)	62 (31.8)	133 (68.2)		
Frequency of using moisturizer					
<3 times/d	295 (35.6)	107 (36.3)	188 (63.7)	35.17	<0.001
3-5 times/d	278 (33.5)	75 (27.0)^c^	203 (73)		
6-10 times/d	146 (17.6)	19 (13.0)^c^	127 (87.0)		
>10 times/d	110 (13.3)	17 (15.5)^c^	93 (84.5)		
Duration of using rubber gloves					
Never	30 (3.6)	9 (30.0)	21 (70.0)	4.615	0.099
0.5-1 hour/d	432 (52.1)	100 (23.1)	332 (76.9)		
More than 1 hour/d	367 (44.3)	109 (29.7)	258 (70.3)		

Risk factor analysis for HE

Nursing staff were divided into four groups based on the frequency of daily handwashing. There were statistically significant differences in HE incidence among these groups (χ^2^ = 7.880, p = 0.049). Specifically, the group washing hands 0-5 times per day had a lower incidence (12.1%) compared to those washing 6-10 times per day (26.3%, χ^2^ = 5.095, p = 0.024), 11-20 times per day (25.9%, χ^2^ = 5.157, p = 0.023), and >20 times per day (29.9%, χ^2^ = 7.745, p = 0.005). No significant differences were observed among the latter three groups (p > 0.05).

Based on the frequency of daily contact with soap/hand sanitizer, participants were divided into three groups, with statistically significant differences in HE incidence (χ^2^ = 13.46, p = 0.001). The group with <10 contacts per day had a lower incidence (18.6%) compared to those with 11-20 contacts per day (26.1%, χ^2^ = 4.398, p = 0.036) and >20 contacts per day (34.1%, χ^2^ = 13.409, p < 0.001). There was also a significant difference between the latter two groups (χ^2^ = 4.33, p = 0.037).

Participants were divided into four groups based on the frequency of daily use of hand moisturizer, with statistically significant differences in HE incidence (χ^2^ = 35.17, p < 0.001). The group using moisturizer > 10 times per day had a lower incidence (15.5%) compared to those using it 3-5 times per day (27.0%, χ^2^ = 5.786, p = 0.016) and <3 times per day (36.3%, χ^2^ = 16.344, p < 0.001). No significant difference was observed between the group using moisturizer > 10 times per day and those using it 6-10 times per day (13.0%, χ^2^ = 0.309, p = 0.578). No statistically significant differences in HE incidence were found among groups divided based on the frequency of daily contact with alcohol-based hand rub or the duration of rubber glove use (p > 0.05) (Table 2).

The incidence of allergic rhinitis, allergic conjunctivitis, asthma, and metal allergy (i.e., rash after contact with metal buttons, metal fasteners, metal jewelry, or other metal objects near the skin) was higher in the HE group than in the non-HE group (p < 0.05). The incidence of dry skin was also higher in the HE group (28.44% vs. 7.03%, χ^2^ = 66.537, p < 0.001). Binary logistic regression analysis indicated that age, work experience, presence of allergic rhinitis, allergic conjunctivitis, metal allergy, frequency of daily handwashing, and frequency of contact with soap/hand sanitizer were risk factors for HE, while the use of hand care products was a protective factor (Table 3).

**Table 3:** Logistic regression analysis of risk factors for hand eczema OR: odds ratio; CI: confidence interval

	OR (95% CI)	p-value
Age	1.029 (1.008~1.049)	0.006
Gender	2.795 (0.831~9.404)	0.097
Work experience	1.03 (1.01~1.05)	0.003
Allergic rhinitis	3.015 (2.144~4.238)	<0.001
Allergic conjunctivitis	5.601 (3.258~9.63)	<0.001
Asthma	2.56 (0.975~6.722)	0.056
Metal allergy	4.865 (2.827~8.370)	<0.001
Frequency of handwashing	1.224 (1.027~1.458)	0.024
Frequency of contact with soap/hand sanitizer	1.52 (1.22~1.89)	<0.001
Frequency of using alcohol-based hand sanitizer	1.15 (0.99~1.33)	0.068
Frequency of using skin care products	0.626 (0.528~0.742)	<0.001
Duration of using rubber gloves	1.27 (0.96~1.68)	0.092

## Discussion

HE is strongly associated with certain occupations, particularly those involving frequent contact with water, detergents, and liquid metals. Nurses constitute one of the high-risk groups for HE. The high incidence of HE among nursing staff is primarily attributed to wet work exposure, usage of protective gloves during work, and AD [4], findings that are consistent with our study. Specifically, age, work experience, daily frequency of handwashing, and frequency of contact with soap/hand sanitizer, as well as the presence of allergic rhinitis, allergic conjunctivitis, and metal allergies, were identified as risk factors for HE. Among nurses, the occupational allergens most often cited are metals (especially nickel and cobalt salts), rubber chemicals, preservatives and disinfectants (such as formaldehyde, glutaraldehyde, chlorhexidine, and mercury compounds), fragrances, and pharmaceutical agents including antibiotics, local anesthetics, and essential oils [5-8]. Beyond significantly impacting patients' daily lives and work, HE can also lead to an increased carriage of potential skin microbes at the affected sites, thereby raising the risk of bacterial infection and transmission. Studies have shown that HE can increase the risk of methicillin-resistant *Staphylococcus aureus* (MRSA) colonization among nursing staff [9].

Numerous studies have investigated the incidence of HE among nursing staff, revealing significant variations across different regions and departments. Reports from Norway, Sweden, and Denmark indicate a prevalence of 20% among healthcare workers [10-13], markedly higher than the general population (14.5%) [3]. In contrast, Japan reported a prevalence of 53% [14], and the prevalence rate of self-reported HE was 31.0% and symptom-based 75.6% in Korea [5]. An epidemiological survey found that the prevalence of HE among nursing staff in Harbin, China, was 20% [15], while in Hong Kong, the prevalence was 22.1% [16]. Since the outbreak of COVID-19, changes in hand hygiene practices and the usage of protective equipment have led to a noticeable increase in the prevalence of HE among healthcare workers. For instance, the incidence of HE among nursing interns during the pandemic was reported to be as high as 49% [4]. A survey of 526 frontline medical staff combating COVID-19 revealed that 74.5% had experienced hand skin damage, with those washing their hands more than 10 times a day being at a greater risk [17]. In our study, the prevalence of HE was 26.3%, with no significant difference in incidence between men and women or between ICU and non-ICU departments. However, the incidence in surgical departments was significantly higher than in non-surgical departments (χ^2^ = 3.951, p = 0.047).

Frequent handwashing and contact with soap/hand sanitizer can disrupt the skin barrier function and affect the diversity and composition of the skin microbiota. The use of moisturizers is an essential measure for the treatment and prevention of HE. Moisturizers can repair the skin barrier, prevent dry skin and fissures, and lower the levels of *S. aureus* in the skin of patients with atopic constitutions [18]. Our study found that the use of moisturizers is a protective factor against HE. It is recommended to apply hand cream after each handwashing; a lipid-rich moisturizer should be preferred, yet no ingredient has proven superior. Choice should be tailored to each patient’s sensitivities, preferences, and budget, and fragrance-free formulas are advised to minimize allergy risk [1,19]. The Expert Consensus on Skin and Mucosal Barrier Protection for Medical Staff Fighting COVID-19 [20] mentioned that 12.4% of medical staff wore three layers of gloves simultaneously during their routine work. Prolonged glove use can lead to excessive hydration of the stratum corneum, causing maceration and erosion; the chemicals in latex gloves can easily cause contact dermatitis, corrode the skin, and lead to secondary infections. Our study found that as the frequency of handwashing and contact with soap/hand sanitizer increased, so did the incidence of HE. However, no significant association was found between the duration of rubber glove use and the occurrence of HE. This may be due to the small sample size.

The etiology of HE is complex, involving not only external factors but also internal factors like atopic constitution. Based on etiology, HE can be categorized into three types: irritant contact dermatitis caused by contact with irritants, allergic contact dermatitis caused by allergens, and hand dermatitis occurring on the basis of atopic constitution. Patients with AD have primary or secondary impairment of skin barrier function, increased skin permeability, and heightened sensitivity to various irritants. A nationwide population-based study in Denmark showed that AD is linked to contact sensitization and a higher prevalence of HE [21]. People who have both AD and HE tend to experience more severe disease symptoms. Studies have shown that approximately 60% of AD patients have hand involvement [22], 34.4% of adult HE patients have a history of AD [3], and up to 25% of patients with moderate to severe AD in childhood develop varying degrees of HE in adulthood [23]. Currently, HE and AD are considered to be the result of skin barrier damage and immune dysregulation caused by various internal and external factors [24]. Although our study did not investigate the prevalence of AD, we found that the incidence of atopic diseases such as allergic rhinitis, allergic conjunctivitis, and metal allergies was significantly higher in the HE group than in the non-HE group, and the incidence of dry skin was also higher. In this study, 27.8% of participants reported itching in flexural areas that had persisted for at least six months. Logistic regression analysis showed that allergic rhinitis, allergic conjunctivitis, and metal allergies are risk factors for HE, further confirming the close relationship between AD and HE.

The limitations of this study are as follows. First, the sample size is relatively small, with only nursing staff from hospitals in certain areas of Xinjiang selected as subjects, which may not be representative of nursing staff across the entire Xinjiang region or beyond. Second, the study relies on questionnaire surveys, which may be subject to recall bias and inaccuracies of self-reported data.

## Conclusions

Our study analyzed the prevalence and influencing factors of HE among nursing staff in certain regions of Xinjiang during the COVID-19 pandemic and found that hand hygiene practices and atopic constitution are the main factors affecting the onset of HE. Additionally, the climate of Xinjiang, characterized by its inland location, dryness, scarce rainfall, and cold winters, may contribute to the incidence of HE. Patients with HE require comprehensive management, including the removal or avoidance of causative factors, behavioral changes, and social support. It is recommended that individuals engaged in nursing or wet work, especially those with atopic constitutions, undergo targeted primary interventions, including standardizing handwashing schedules, reducing the use of alkaline handwash, and strengthening health education and training in skin care and self-protection, to lower the incidence of HE.

## Appendices

**Table 4:** Questionnaire of the study

1.	Unit:					
2.	Department:					
3.	Gender:	Male	Female			
4.	Professional years: How many years have you been working?	_____ years				
5.	Have you ever had hand eczema?	No _____	Yes _____			
6.	Do you have a history of allergic rhinitis?	No _____	Yes _____	Don't know _____		
7.	Do you have a history of allergic conjunctivitis?	No _____	Yes _____	Don't know _____		
8.	Do you have a history of asthma? Was it diagnosed by a doctor?	No _____ No _____	Yes _____ Yes _____	When? _____ year		
9	How many times do you wash your hands on a regular workday?	A. 0-5 times	B. 6-10 times	C. 11-20 times	D. More than 20 times	
10	How many times do you come into contact with soap/hand wash daily?	A. Less than 10 times	B. 11-20 times	C. More than 20 times		
11	How many times do you use alcohol-based hand sanitizer daily?	A. 0-5 times	B. 6-10 times	C. 11-20 times	D. More than 20 times	
12	How many times do you use hand care products daily?	A. Less than 3 times	B. 3-5 times	C. 6-10 times	D. More than 10 times	
13	On average, how many hours do you use rubber gloves daily?	A. Never use	B. 0.5-1 hour	C. More than 1 hour		
14	On average, how much time do you spend cooking daily?	A. 0	B. Less than half an hour	C. More than half an hour		
15	On average, how much time do you spend cleaning or washing clothes daily?	A. 0	B. Less than half an hour	C. More than half an hour		
16	On average, how much time do you spend taking care of children under 4 years old daily?	A. 0	B. Less than half an hour	C. More than half an hour		
17	Have you ever experienced skin symptoms due to wearing protective gloves? What type of gloves?	No _____ Natural rubber/latex _____	Yes _____ Synthetic rubber _____	Plastic _____	Leather _____ Other _____ Don't know _____	
18	Have you ever changed the type of gloves or stopped using gloves due to skin symptoms?	No _____	Yes _____ When? _____ (year)			
19	Have you ever had a history of urticaria (itchy wheals that appear and disappear rapidly within hours)?	A. No (go to Question 32)	B. Yes			
20	How many times have you had urticaria?	A. Once	B. 2-5 times	C. More than 5 times		
21	When was the last time you had these itchy wheals (urticaria)?	A. Within the past 7 days	B. 7 days to 3 months	C. 3-12 months	D. More than 1 year ago In which year? _____ (year)	
22	When did you first have these itchy wheals (urticaria)?	A. Under 6 years old	B. 6-14 years old	C. 15-18 years old	D. Over 18 years old In which year did it start? _____ (year)	
23	What was your occupation when you first had urticaria?					
24	Have you ever seen a doctor for wheals (urticaria) since adulthood?	No _____	Yes _____ When was the last time? _____ (year)			
25	In the past 12 months, have you had any of the following symptoms on your hands? A. No symptoms in the past 12 months B. Redness C. Dry skin, with scales D. Cracks E. Exudation or crusting F. Tiny blisters G. Papules H. Rapid appearance of itchy wheals/lumps (urticaria) I. Itching J. Burning, stinging K. Tenderness L. Pain Other	No _____	Yes _____			
26	Have you ever had a rash due to metal buttons, metal fasteners, metal jewelry (e.g., earrings), or other metal objects near your skin?	A. No	B. Yes			
27	Is your skin often dry?	A. No	B. Yes			
28	Does your skin itch when you sweat?	A. No	B. Yes			
29	Has a doctor ever diagnosed you with an allergy?	No _____ (go to Question 31)	Yes _____	Don't know _____ (go to Question 31)		
30	Do your parents have any allergic diseases?	No _____	Yes _____ A. Urticaria B. Asthma C. Allergic rhinitis D. Other			
31	How were you diagnosed with an allergy?	A. Patch test	B. Skin scratch test	C. Blood test	D. Other ________________	
32	Have you ever had persistent skin itching for at least 6 months, sometimes affecting skin folds? (We are referring to skin folds such as elbow creases, behind the knees, front of the ankles, under the buttocks, neck, around the ears or eyes)	A. No	B. Yes	C．Don’t know		
33	Have you ever had eczema on your wrists or forearms?	No _____ (If "No," no need to answer the following questions)	Yes _____			
34	How often do you have eczema on your hands?	A. Just once and for less than 2 weeks	B. Only once but lasting more than 2 weeks	C. More than once		
35	Please draw the areas on your hand or forearm where eczema often occurs (one or more areas)					
36	When was the last time you had hand eczema?	A. Just now	B. Within the past 3 months	C. 3-12 months ago	D. More than 12 months ago In which year was the last time? _____ (year)	
37	When did you first have eczema on your hands, wrists, or forearms?	A. Under 6 years old	B. 6-14 years old	C. 15-18 years old	D. Over 18 years old In which year did it start? _____ (year)	
38	What do you think caused the eczema on your hands, wrists, or forearms when it first started?					
39	In which season do you have the most problems with hand eczema?	A. No seasonal difference	B. Winter	C. Spring	D. Summer	E. Autumn
40	Have you noticed that contact with certain materials, chemicals, or anything else at work makes your eczema worse?	A. No	B. Yes What is it? ____________________________	C. Don't know		
41	Have you noticed that contact with certain materials, chemicals, or anything else outside of work makes your eczema worse?	A. No	B. Yes What is it? ____________________________			
42	Do you think the most important things outside the workplace that exacerbate your eczema are A. Soap, liquid soap, shampoo, and other personal hygiene products B. Detergents and other household cleaning and laundry products C. Food handling D. Working with wet hands E. Frequent hand washing F. Protective gloves G. Machine maintenance (e.g., automobiles), handling oil H. Construction work, paint, wallpaper, renovation, and decoration I. Gardening, handling plants, soil, vegetables, berries, fruits, etc. J. Infections (cold, flu, or fever) K. Mood, stress L. Menstrual period or other hormonal factors M. Other ________________________________					
43	Does your eczema improve when you are away from your regular job (e.g., weekends or longer periods)?	A. No	B. Yes			
